# Implementation and Outcome of Robotic Liver Surgery in the Netherlands: A Nationwide Analysis

**DOI:** 10.1097/SLA.0000000000005600

**Published:** 2022-07-18

**Authors:** Burak Görgec, Maurice Zwart, Carolijn L. Nota, Okker D. Bijlstra, Koop Bosscha, Marieke T. de Boer, Roeland F. de Wilde, Werner A. Draaisma, Michael F. Gerhards, Mike S. Liem, Daan J. Lips, Hendrik A. Marsman, J. Sven D. Mieog, Quintus I. Molenaar, Maarten Nijkamp, Wouter W. Te Riele, Türkan Terkivatan, Alexander L. Vahrmeijer, Marc G. Besselink, Rutger-Jan Swijnenburg, Jeroen Hagendoorn

**Affiliations:** * Amsterdam UMC, location University of Amsterdam, Department of Surgery, Amsterdam, the Netherlands; † Cancer Center Amsterdam, Amsterdam, the Netherlands; ‡ Department of Surgery, University Medical Center Utrecht, Utrecht, the Netherlands; § Department of Surgery, Leiden University Medical Center, Leiden, the Netherlands; ∥ Department of Surgery, Jeroen Bosch Hospital, ‘s-Hertogenbosch, the Netherlands; ¶ Department of Surgery, University Medical Center Groningen, Groningen, the Netherlands; # Department of Surgery, Erasmus MC Cancer Institute, Erasmus University Medical Center, Rotterdam, the Netherlands; ** Department of Surgery, OLVG, Amsterdam, the Netherlands; †† Department of Surgery, Medical Spectrum Twente, Enschede, the Netherlands; ‡‡ Department of Surgery, St. Antonius Hospital, Nieuwegein, the Netherlands; §§ Amsterdam UMC, location Vrije Universiteit Amsterdam, Department of Surgery, Amsterdam, the Netherlands

**Keywords:** hepatic resection, implementation; liver resection, liver surgery, minimally invasive liver resection; minimally invasive liver surgery, nationwide analysis; robotic liver resection; robotic liver surgery

## Abstract

To determine the nationwide implementation and surgical outcome of minor and major robotic liver surgery (RLS) and assess the first phase of implementation of RLS during the learning curve.

**Background:**

RLS may be a valuable alternative to laparoscopic liver surgery. Nationwide population-based studies with data on implementation and outcome of RLS are lacking.

**Methods:**

Multicenter retrospective cohort study including consecutive patients who underwent RLS for all indications in 9 Dutch centers (August 2014–March 2021). Data on all liver resections were obtained from the mandatory nationwide Dutch Hepato Biliary Audit (DHBA) including data from all 27 centers for liver surgery in the Netherlands. Outcomes were stratified for minor, technically major, and anatomically major RLS. Learning curve effect was assessed using cumulative sum analysis for blood loss.

**Results:**

Of 9437 liver resections, 400 were RLS (4.2%) procedures including 207 minor (52.2%), 141 technically major (35.3%), and 52 anatomically major (13%). The nationwide use of RLS increased from 0.2% in 2014 to 11.9% in 2020. The proportion of RLS among all minimally invasive liver resections increased from 2% to 28%. Median blood loss was 150 mL (interquartile range 50–350 mL] and the conversion rate 6.3% (n=25). The rate of Clavien-Dindo grade ≥III complications was 7.0% (n=27), median length of hospital stay 4 days (interquartile range 2–5) and 30-day/in-hospital mortality 0.8% (n=3). The R0 resection rate was 83.2% (n=263). Cumulative sum analysis for blood loss found a learning curve of at least 33 major RLS procedures.

**Conclusions:**

The nationwide use of RLS in the Netherlands has increased rapidly with currently one-tenth of all liver resections and one-fourth of all minimally invasive liver resections being performed robotically. Although surgical outcomes of RLS in selected patient seem favorable, future prospective studies should determine its added value.

The use of minimally invasive liver surgery increased gradually in the last 3 decades.^1^ Concerns about technical difficulties combined with long learning curves have hampered the adoption of minimally invasive liver surgery.^1^ A recent study proposed a 3 phase model of learning curves including a competency (operative time, blood loss, and conversion), proficiency (morbidity, mortality, and hospital stay), and mastery phase (textbook- or benchmark outcomes).^2^ Nevertheless, laparoscopic liver surgery is currently available in most centers, with several reports showing its advantages compared with open liver surgery, including reduced intraoperative blood loss, less transfusions, fewer complications, and a shorter hospital length of stay.^1^,^3^–^10^ According to the international Southampton guidelines, laparoscopy is now seen as the standard for minor liver resections.^11^ Major laparoscopic liver resections should be implemented in a stepwise manner and combined with structured training in centers who have completed the learning curve for minor laparoscopic liver resections.^7^,^8^,^11^–^14^ A recent nationwide study in the Netherlands showed an overall good adherence to this concept with a steady increase of the proportion of technically major and anatomically major laparoscopic liver resections over the years.^15^

Robotic liver surgery (RLS) represents the most recent evolution in the field of minimally invasive liver surgery and has been suggested as a valuable alternative to laparoscopic liver surgery. Perceived benefits of RLS include a better magnified 3-dimensional view, articulating instruments, tremor filtration, platform for image-guided surgery, ease of suturing, improved ergonomics, and better motion scaling, as compared with the laparoscopic approach.^16^–^19^ Still, the widespread diffusion of RLS is limited, potentially due to higher cost and suboptimal availability of robotic systems. Several high-volume expert centers have shown the potential advantages of RLS as compared with open liver surgery, whereas other centers are still exploring the use of robotics for liver surgery.^20^–^24^

Recently, several Dutch centers have implemented RLS into their daily surgical practice. Nationwide population-based studies with data on both implementation and surgical outcome of RLS are lacking. The aim of this study is to determine the rate of implementation and surgical outcome of minor and major RLS on a nationwide scale and assess the first phase of implementation of RLS including the learning curve.

## METHODS

A multicenter retrospective cohort study was performed to provide insights in the implementation rates and surgical outcome of RLS in the Netherlands. Data were gathered from all 9 liver surgical centers in the Netherlands with an RLS program: Amsterdam UMC, Amsterdam; Erasmus University Medical Center, Rotterdam; Leiden University Medical Center, Leiden; University Medical Center Groningen, Groningen; OLVG, Amsterdam; Medical Spectrum Twente, Enschede; Jeroen Bosch Hospital, ‘s-Hertogenbosch; St. Antonius Hospital, Nieuwegein; and University Medical Center Utrecht, Utrecht.

The study was initiated by the Dutch Liver Collaborative Group (DLCG) and reported in compliance with the Strengthening the Reporting of Observational Studies in Epidemiology (STROBE) statement.^25^ All data were handled anonymously. Hence, the ethics committee of the Amsterdam UMC assessed that the current study was not subject to the Medical Research Involving Human Subjects Act and waived the need for informed consent.

### Data Source and Patient Selection

Data on all liver resections including laparoscopic and open liver resections in all 27 centers performing liver surgery in the Netherlands were obtained from the Dutch Hepato Biliary Audit (DHBA) to assess nationwide implementation rates of RLS. The DHBA is a nationwide prospective registry in which all Dutch hospitals performing liver surgery are obliged to record all types of liver resections performed.

Patients after RLS were initially identified using center specific liver surgery databases and individual patient data were extracted from the DHBA.

Data of all consecutive patients who underwent RLS for all indications between January 2014 and March 2021 were included. Patients were excluded when no formal resection was performed (such as fenestration/deroofing of cysts and biopsies) or in case of emergency surgery.

### Definitions and Outcomes

Minor liver resection was defined as any resection from the anterolateral segments, that is, 2, 3, 4b, 5, and 6. Anatomically major liver resection was defined as resection of 3 or more Couinaud’s segments.^26^ Technically major liver resection was defined as any resection from the posterosuperior segments, that is, 7, 8, 4a, and 1. The Kawaguchi difficulty scoring system was calculated per patient and defines 3 groups of difficulty based on the type of resection: group I (low) includes wedge resection and left lateral sectionectomy, group II (intermediate) includes anterolateral segmentectomy and left hepatectomy, and group III (high) includes posterosuperior segmentectomy, right posterior sectionectomy, right hepatectomy, central hepatectomy, and extended left/right hepatectomy.^27^

Baseline characteristics consisted of age, sex, body mass index (kg/m^2^), American Society of Anesthesiologists (ASA) grade, Charlson Comorbidity Index, cirrhosis, neoadjuvant chemotherapy, previous extrahepatic abdominal surgery, previous liver surgery, histologic diagnosis, number of lesions, size of largest lesion, distribution of lesions (ie, uni- or bilobar), and extent of resection.

Surgical outcomes included intraoperative blood loss (defined by the measured amount of suctioned blood with 20 mL increments), conversion to laparotomy, 30-day overall postoperative complications (defined according to the Clavien-Dindo classification^28^ and Comprehensive Complication Index^29^), severe postoperative complications (defined as Clavien-Dindo ≥3^28^), 30-day readmission, 30-day reoperation, postoperative length of hospital stay (LOS), R0 resection margin (ie, 1 mm or more tumor free margin), and 30-day or in-hospital mortality.

### Survey

A short survey was developed using Google Forms Survey (Google; Mountain View, CA) and was disseminated by email to all local study investigators of the nine participating centers (Supplement 1, Supplemental Digital Content 1, http://links.lww.com/SLA/E50). The survey included questions with regards to form of individual training surgeons completed before start with their RLS program, surgical technique, intraoperative management, and case selection for RLS.

### Statistical Analysis

Data were analyzed using IBM SPSS Statistics for Windows version 25.0 (SPSS Inc., Chicago, IL). Continuous, not normally distributed variables were expressed as median with interquartile range (IQR). In case variables were normally distributed, they were reported as mean with SD. A Mann-Whitney U test was used to compare continuous, not normally distributed variables between groups. Normally distributed, continuous variables were compared using an independent samples *t* test. Categorical variables were reported as frequencies and proportions and compared between groups using a χ^2^ test.

Outcomes were stratified into type of resection (minor, technically major, and anatomically major RLS) to better understand outcome distribution. Trends in time were explored by dividing patients into the first 200 and second 200 RLS procedures. A sensitivity analysis was performed by stratifying patients according to the Kawaguchi difficulty scoring system into 3 groups to be able to compare outcomes with previous literature. A subgroup analysis included stratification for centers where the leading console surgeon did nor did not complete a fellowship in minimally invasive liver surgery. A second subgroup analysis included stratification for liver cirrhosis. Correlations were expressed in Spearman’s Rho with *P* value.

The learning curve effects on blood loss and conversion (phase of competency), and major morbidity and hospital stay (phase of proficiency) were assessed with trends over consecutive procedures per center with cumulative sum (CUSUM) analyses. The learning curves were assessed overall and, if a significant correlation was found, on multivariate analysis taking center and minor or major resection into account. First, the patients were ranked consecutively according to the date of their procedure and the difference of the data to the mean per center was calculated per case. Hereafter, the data were aggregated for all centers with a weighting for the volume of resections and a CUSUM was presented on the *y* axis per case. The magnitude by which the line ascends or descends is determined by the difference between the observed and expected outcome. For example, the line ascends when blood loss in that case was above average for that center by an amount relative to the SD, and for a case where blood loss was below average, the line descends. The top of the CUSUM graph thus represented the total blood loss in SD above average up to that case.

## RESULTS

### Nationwide Implementation of RLS

**FIGURE 1:**
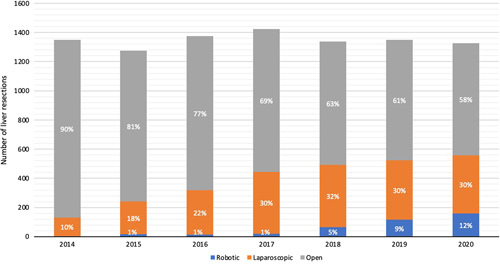
Annual rate of open, laparoscopic, and robotic liver surgery in the Netherlands (2014–2020).

**FIGURE 2:**
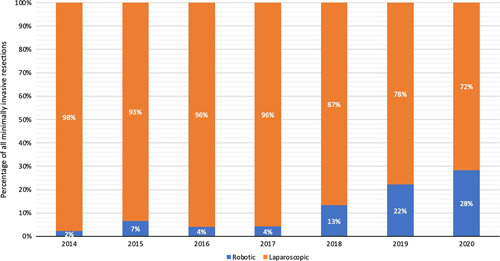
Annual proportion of robotic and laparoscopic liver surgery in all patients after minimally invasive liver surgery in the Netherlands (2014–2020).

**FIGURE 3:**
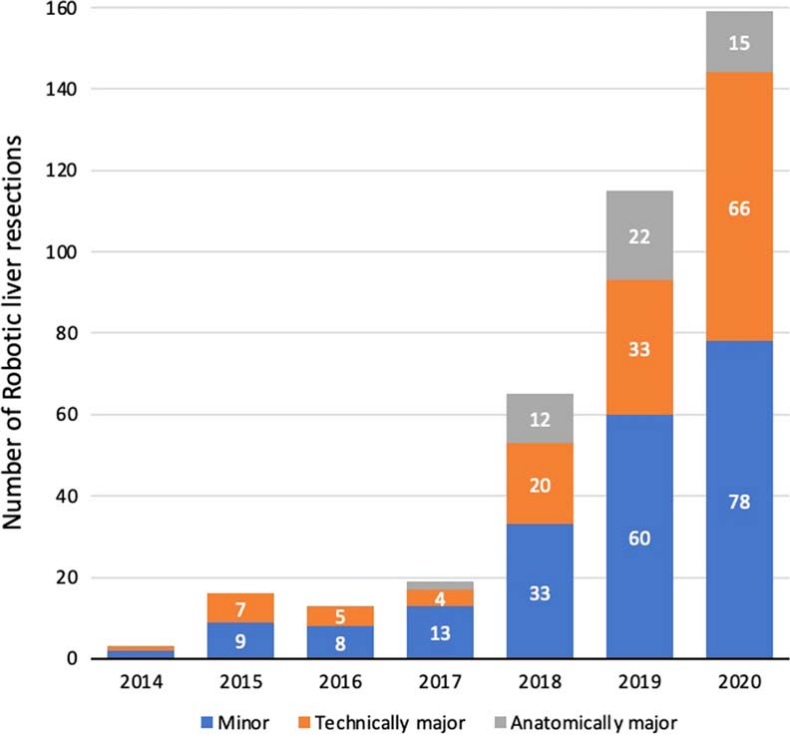
Annual volume of robotic liver resections stratified for type of resection.

During the 7-year study period, a total of 9437 liver resections were performed in 27 centers in the Netherlands including 2320 laparoscopic liver resections (24.6%) and 400 RLS (4.2%) procedures. The RLS procedures were performed in 9 of the 27 centers. The nationwide use of RLS per year increased from 3 patients (0.2%) in 2014 to 158 patients (11.9%) in 2020 (Fig. 1) (*P*<0.001). Within the group of minimally invasive liver resections, the annual use of RLS increased from 2% in 2014 to 28% in 2020 (*P*<0.001) (Fig. 2). Figure 3 shows the annual volume of RLS categorized by type of resection.

### Center Characteristics

Five out of the 9 centers that performed RLS were university medical centers, whereas 4 centers were large teaching hospitals. A total of 19 surgeons performed RLS with a median of 2 surgeons (IQR 2-3) per center. A gradual implementation of RLS was observed. In 2014, the first Dutch RLS procedure was performed. In 2015, 2 more centers started performing RLS, whereas the remaining 6 centers initiated an RLS program in 2018 (n=4) and 2019 (n=2). The overall median volume of liver surgery per center (including open, laparoscopic, and robotic resections) during the study period was 453 (IQR 255–545) liver resections. The overall median volume of RLS per center was 35 (IQR 23–52) resections with an overall median implementation rate per center of 10.2% ranging from 2.1% to 30.8% between centers. The mean annual volume of RLS per center in 2018 was 7 (range 0–12), which increased to 18 (range 7–31) in 2020. The annual use of RLS in the nine centers increased from 0.5% in 2014 to 26% in 2020 (*P*<0.001), whereas the annual use of laparoscopic liver surgery increased from 14.9% in 2014 to 26% in 2017 before reducing to 13% in 2020 (Supplement 2, Supplemental Digital Content 1, http://links.lww.com/SLA/E50). Overall, the use of minimally invasive liver surgery (combining RLS and laparoscopic liver surgery) in the 9 centers increased from 15.4% (n=95) in 2014 to 29.0% (n=241) in 2020 (*P*<0.001).

### Surgeon Experience and Training

In 7 of the 9 centers, experience was obtained with robotic abdominal surgery before (n=4; 44%) or in parallel with (n=3; 33%) the start of performing RLS. Experience in previous robotic abdominal surgery in the 4 centers included robotic cholecystectomy and colorectal resections.

The 9 leading robotic console surgeons from the participating centers indicated that their training in RLS consisted of the Intuitive Surgical basic robotic surgery course (n=7; 78%), observership programs in an expert robotic liver surgical center (n=7; 78%) ranging from 1 day to 6 weeks, proctorship programs in own center provided by an international proctor robotic liver surgeon (n=5; 56%), fellowships in minimally invasive HPB surgery (n=3; 33%) ranging from 1 to 2.5 years, and hands-on courses of 1 or 2 days in minimally invasive liver surgery (n=4; 44%).

### Patient Selection for RLS

The nine leading console surgeons described several patient-related factors used by the individual center to select patients for RLS. Overall, reported selection factors for RLS were absence of centrally located tumors (3 of 9 centers; 33%), no indication for technically or anatomically major liver resection (3 of 9 centers; 33%), absence of major vascular or biliary duct involvement (2 of 9 centers; 22%), small lesions (2 of 9 centers; 22%), absence of perihilar cholangiocarcinoma (3 of 9 centers; 33%) or gallbladder carcinoma (1 of 9 centers; 11%), absence of liver cirrhosis (1 of 9 centers; 11%), no indication for an extended hemihepatectomy (2 of 9 centers; 22%), and absence of extensive previous abdominal surgery (1 of 9 centers; 11%). The selection factors for RLS per center are presented in Supplement 3, Supplemental Digital Content 1, http://links.lww.com/SLA/E50.

### Surgical Technique

Surgical techniques among the participating centers were largely comparable for minor and major RLS and are displayed in Supplement 4, Supplemental Digital Content 1, http://links.lww.com/SLA/E50. In 7 centers (78%), RLS was performed using the da Vinci Xi Robotic Surgical System (Intuitive Surgical^®^, Inc., Sunnyvale, CA), whereas in 2 centers (22%), the da Vinci X Robotic Surgical System was used. Specimens were extracted in a plastic endoscopic bag through a widened trocar incision in case of small lesions, or a Pfannenstiel incision in case of larger lesions or anatomically major resections.

### Baseline Characteristics

A total of 400 patients after RLS met the inclusion criteria and were included in the analysis. Baseline characteristics including a stratification for type of resection are shown in Supplement 5, Supplemental Digital Content 1, http://links.lww.com/SLA/E50. Median age was 64 years (IQR 53.0–72.0) and 179 patients (44.7%) were women. Most patients were an American Society of Anesthesiologists 1 or 2 (n=286; 72.2%) with a median Charlson Comorbidity Index of 3 (IQR 2–5). Neoadjuvant chemotherapy was applied in 66 patients (16.5%), whereas the majority of patients had undergone previous abdominal surgery (n=239; 61.4%). On histologic diagnosis, malignant lesions were observed in 333 patients (84.5%). Median lesion size was 27.0 mm (IQR, 17.0–43.0). Most patients underwent a minor RLS (n=207; 51.8%) followed by 141 patients (35.3%) who underwent a technically major RLS and 52 patients (13%) who underwent an anatomically major RLS.

### Surgical Outcome

Table 1 shows the surgical outcome after all RLS and stratified for minor, technically major, and anatomically major RLS. The overall conversion rate was 6.3% and ranged from 3.9% in the minor RLS group to 9.6% in the anatomically major RLS group. Severe postoperative complications occurred in 27 patients (7.0%) of the total cohort (5.4% after minor, 7.4% after technically major, and 12.5% after anatomically major RLS). The 3 main severe postoperative complications were bile leakage (1.3%), intra-abdominal abscess (1.3%) and hemorrhage (0.8%). The overall Comprehensive Complication Index was 4.6 (12.3) with 3.9 (10.2) in the minor RLS group, 4.2 (12.5) in the technically major RLS group and 8.6 (18.2) in the anatomically major RLS group. Overall hospital stay was 4 days (IQR 2–5 d) with a 30-day mortality rate of 0.8%. Radical resection (R0 resection margin) in case of malignancy was achieved in 264 patients (83.3%) varying from 136 patients (86.6%) in the minor RLS group to 26 patients (70.3%) in the anatomically major RLS group. Outcomes stratified for the Kawaguchi difficulty scoring system are shown in Supplement 6, Supplemental Digital Content 1, http://links.lww.com/SLA/E50. Stratifying outcomes for fellowship training showed that although more technically and anatomically major resections were performed by fellowship-trained surgeons, outcomes were largely comparable except the rate of overall complications (10.8% vs 23.0%; *P*=0.005) (Supplement 7, Supplemental Digital Content 1, http://links.lww.com/SLA/E50). Stratifying outcomes for liver cirrhosis showed that although outcomes tended to be inferior in patients with cirrhosis as compared with patients without cirrhosis, there we no significant differences between outcomes except mortality (6.9% vs 0.3%; *P*<0.001) (Supplement 8, Supplemental Digital Content 1, http://links.lww.com/SLA/E50).

**TABLE 1:** Surgical Outcome After Robotic Liver Resection, Stratified for Type of Surgery

	All RLS N=400	Minor RLS N=242	Technically Major RLS N=141	Anatomically Major RLS N=52
Blood loss (mL), median (IQR)	150 (50–350)	100 (24–200)	200 (100–500)	300 (150–1200)
Conversion to laparotomy	25 (6.3)	8 (3.9)	12 (8.5)	5 (9.6)
Postoperative complications	76 (19.1)	37 (17.9)	23 (16.3)	16 (31.4)
Severe postoperative complications	27 (7.0)	11 (5.4)	10 (7.4)	6 (12.5)
CCI, mean (SD)	4.6 (12.3)	3.9 (10.2)	4.2 (12.5)	8.6 (18.2)
Postoperative hospital stay (d), median (IQR)	4 (2–5)	3 (2–3)	3 (2–5)	6 (4–10)
Reoperation within 30 d	10 (2.6)	5 (2.5)	4 (2.9)	1 (2.1)
Readmission within 30 d	12 (3.0)	4 (1.9)	5 (3.6)	3 (6.0)
R0 resection in case of malignancy	264 (83.3)	136 (86.6)	102 (82.9)	26 (70.3)
30-d mortality	3 (0.8)	0	1 (0.7)	2 (3.9)

Values in parentheses are percentages unless mentioned otherwise.

CCI indicates Comprehensive Complication Index; mL, milliliter.

### Trends in Time

Baseline characteristics and operative outcomes of the first and second 200 consecutive RLS procedures across the study period are shown in Tables 2 and 3, respectively. In the second 200 RLS procedure more patients received neoadjuvant chemotherapy (12.5% vs 20.5%; *P*=0.031) and more patients had previous extrahepatic abdominal surgery (44.9% vs 55.5%; *P*=0.038) as compared with the first 200 procedures. There was no significant difference in histologic diagnosis between both periods (*P*=0.100). In the second 200 procedures, more technically major RLS procedures were performed (28.5% vs. 42.0%; *P*=0.005).

**TABLE 2:** Baseline Characteristics of the First and Second 200 Consecutive Patients After Robotic Liver Surgery

	The First 200 RLS N=200	The Second 200 RLS N=200	*P*
Patient characteristics			
Age, years, median (IQR)	64.0 (50.3–71.0)	64.0 (55.3–73.0)	0.293
Sex, male (%)	105 (52.5)	116 (58.0)	0.269
BMI, kg/m^2^, median (IQR)	26.5 (23.0–30.6)	25.4 (23.0–29.0)	0.067
ASA grade			0.133
ASA 1 (%)	13 (6.6)	20 (10.1)	
ASA 2 (%)	137 (69.5)	116 (58.3)	
ASA 3 (%)	46 (23.4)	62 (31.2)	
ASA 4 (%)	1 (0.5)	1 (0.5)	
Charlston Comorbidity Index, median (IQR)	3 (2–5)	3 (1–6)	0.970
Neoadjuvant chemotherapy (%)	25 (12.5)	41 (20.5)	**0.031**
Cirrhosis (%)	17 (8.5)	12 (6.0)	0.335
Previous extrahepatic abdominal surgery (%)	84 (44.9)	111 (55.5)	**0.038**
Previous liver surgery (%)	16 (8.0)	28 (14.0)	0.057
Tumor characteristics			
Histologic diagnosis			0.100
CRLM (%)	98 (49.0)	120 (61.5)	
HCC (%)	36 (18.0)	18 (9.2)	
Cholangiocarcinoma (%)	6 (3.0)	7 (3.6)	
Gallbladder carcinoma (%)	1 (0.5)	2 (1.0)	
Non-CRLM (%)	11 (5.5)	12 (6.2)	
Other malignancy (%)	12 (6.0)	11 (5.6)	
Benign (%)	36 (18.0)	25 (12.5)	
Number of lesions, median (IQR)	1 (1–2)	1 (1–2)	0.877
Size of largest lesion, mm, median (IQR)	31.0 (20.0–50.0)	25.0 (15.0–40.0)	**0.006**
Distribution of lesions			0.105
Unilobar (%)	151 (89.3)	167 (83.5)	
Bilobar (%)	18 (10.7)	33 (16.5)	
Procedure characteristics			
Type of resection			**0.005**
Minor (%)	109 (54.4)	98 (49.0)	
Technically major (%)	57 (28.5)	84 (42.0)	
Anatomically major (%)	34 (17.0)	18 (9.0)	
Extent of resection			**0.004**
Wedge (%)	69 (34.5)	108 (54.0)	
Segmentectomy (%)	43 (21.5)	34 (17.0)	
Bisegmentectomy (%)	54 (27.5)	40 (20.0)	
Trisegmentectomy	4 (2.0)	1 (0.5)	
Left hemihepatectomy (%)	13 (6.5)	5 (2.5)	
Right hemihepactectomy (%)	15 (7.5)	11 (5.5)	
Extended right hemihepatectomy (%)	2 (1.0)	—	
Other anatomically major (%)	—	1 (0.5)	

Values in parentheses are percentages unless mentioned otherwise. Percentages may not add up due to rounding and missing data.

Bold values indicate statistically significant *P*<0.005.

BMI indicates body mass index; CRLM, colorectal liver metastasis; HCC, hepatocellular carcinoma.

**TABLE 3:** Surgical Outcome After Robotic Liver Resections Stratified for Type of Surgery and for the First and Second 200 Consecutive Patients

	Minor RLS			Technically Major RLS			Anatomically Major RLS		
	First 200 N=109	Second 200 N=98	*P*	First 200 N=57	Second 200 N=84	*P*	First 200 N=34	Second 200 N=18	*P*
Blood loss (mL), median[IQR]	100 (20–165)	100 (30–200)	0.333	300 (100–525)	200 (95–513)	0.620	300 (150–1250)	400 (225–850)	0.829
Conversion to laparotomy	5 (4.6)	3 (3.1)	0.570	5 (8.8)	7 (8.3)	0.927	4 (11.8)	1 (5.6)	0.470
Postoperative complications	24(22.0)	13 (13.3)	0.101	14 (24.6)	9 ((11.0)	**0.034**	11 (32.4)	5 (29.4)	0.830
Severe postoperative complications	7 (6.7)	4 (4.1)	0.407	6 (11.1)	4 (4.9)	0.173	4 (12.9)	2 (11.8)	0.909
CCI, mean (SD)	4.5 (11.1)	3.2 (9.1)	0.351	5.1 (11.7)	3.5 (13.1)	0.474	6.9 (13.2)	11.5 (25.2)	0.407
Postoperative hospital stay (d), median[IQR]	4 (3–5)	3 (2–4)	**<0.001**	4 (3–6)	3 (1.8–4.3)	**<0.001**	7 (5.8–12)	4 (4–6)	**0.002**
Reoperation within 30 d	4 (3.8)	1 (1.0)	0.196	2 (3.7)	2 (2.4)	0.669	1 (3.2)	0	0.454
Readmission within 30 d	3 (2.8)	1 (1.0)	0.366	2 (3.5)	3 (3.7)	0.963	2 (6.1)	1 (5.9)	0.980
R0 resection in case of malignancy	65 (83.3)	71 (89.9)	0.089	43 (84.3)	59 (81.9)	0.731	18 (72.0)	8 (66.7)	0.740
30-d mortality	0	0	1	0	1 (1.2)	0.403	1 (2.9)	1 (5.9)	0.610

Values in parentheses are percentages unless mentioned otherwise. Values in bold are considered statistically significant (*P*<0.05).

CCI indicates Comprehensive Complication Index; mL, milliliter.

Comparing outcomes between the 2 periods stratified for type of resection showed that for all 3 difficulty groups, hospital stay was significantly shorter in the second period as compared with the first period. The remaining outcomes did not differ for the 3 difficulty groups. There was a decreasing trend for hospital stay in each difficulty group (see Supplement 9, Supplemental Digital Content 1, http://links.lww.com/SLA/E50).

### Learning Curve

The blood loss and conversion learning curve was assessed separately for minor and major resections. In the maturation of experience for minor resections, blood loss remained consistent and no decrease of outliers occurred (Rho=−0.031, *P*=0.493). In the maturation of the experience for major resections, blood loss diminished (Rho=−0.231, *P*<0.001). Consequently, CUSUM analysis of blood loss in major resections revealed an inflection point at 33 procedures to a plateau phase till 44 RLS procedures, whereafter blood loss was consistently lower than average (see supplement 10, Supplemental Digital Content 1, http://links.lww.com/SLA/E50). The rate of conversion diminished from 8.3% over the first 10 procedures to 3.2% after the first 30 consecutive procedures of all centers combined (Rho=−0.06, *P*=0.184). The CUSUM curve showed a turning point at 21 procedures for minor resections and 42 procedures for major resections (8% conversion vs 4% conversion, *P*=0.074).

There was no significant correlation between consecutive procedures and severe complication (Rho=−0.071, *P*=0.404) or mortality (Rho=0.077, *P*=0.063) and the CUSUM analysis was inconclusive.

There was a significant decrease in-hospital stay (Rho=−0.091, *P*=0.036), which remained consistent (*P*=0.014) after multivariate analysis adjusting for center and type of resection, *P*=0.318, *P*=0.468, respectively). CUSUM analysis revealed an turning point after 19 procedures for minor resections and after 47 for major resection. Hospital stay was significantly reduced after the turning points *P*=0.043, median 4 [3–6] versus 3 [2–5] days (see supplement 11, Supplemental Digital Content 1, http://links.lww.com/SLA/E50).

Center specific CUSUM analyses of blood loss in major resections stratified for previous laparoscopic liver surgery experience showed a learning curve of 33 major robotic liver resections in centers with previous laparoscopic liver surgery experience and a learning curve of 35 major robotic liver resections in centers without any previous laparoscopic liver surgery experience.

## DISCUSSION

This first nationwide retrospective study on RLS found a remarkable increase in the use of RLS in the Netherlands from 0.2% to 11.9% among all liver resections. Within the group of minimally invasive liver resections, there has been a substantial movement toward robotics, with RLS accounting for over one-fourth of all minimally invasive liver resections in 2020. Intra- and postoperative outcome in these selected patients seem promising with a conversion rate of 6.3%, severe complication rate of 7.0%, 30-day/in-hospital mortality of 0.8%, and a radical oncological resection status of 83.2%. With CUSUM analysis, a learning curve of at least 33 procedures for major RLS was demonstrated.

Nationwide retrospective studies on the implementation and outcomes of RLS are currently lacking and only a few retrospective multicenter studies have been performed. A recent multicenter study (2016–2018) investigated the outcomes of RLS as compared with open and laparoscopic liver surgery in 28 states in the United States including 351 RLS procedures with a 3.1% use of RLS.^30^ The authors reported an overall complication rate of 7.2%, a mean hospital stay of 2 days and a mortality of 0.9%. Although in the current study the overall morbidity was higher and hospital stay longer, the authors did not stratify postoperative outcomes for type of resection. In addition, data regarding blood loss and conversion rates were missing. A second multicenter retrospective study from 5 Italian centers compared the outcome of RLS with laparoscopic liver surgery using propensity score matching and included 403 RLS procedures.^31^ With 12.9% major resections, a conversion rate of 4.8% to 7.9%, severe morbidity of 3.0% to 8.5% and mortality of 0.5% depending on the Kawaguchi difficulty level of the RLS procedure, their results are comparable with the outcomes of the current study. Of note, this study did not focus on nationwide implementation and only included high-volume expert centers. The 7% rate of severe postoperative complications in the current study was lower than the 11.1% reported in the overall DHBA cohort.^32^ Of note, outcomes in that study were not stratified for surgical approach. The R0 resection margin in the current study was lower than reported in previous studies, especially for anatomically major liver resections.^30^,^31^ Also, the rate of R0 resection rate in the current robotic cohort was lower than the overall R0 resection rate in the DHBA, regardless of surgical approach.^33^ It has been suggested that in certain patients with colorectal liver metastases and hepatocellular carcinoma, R1 resection may be inevitable and should not be considered directly as a technically error, especially in case of R1 vascular resection.^34^–^36^ Nevertheless, the R0 rate in the current study clearly shows that anatomically major robotic liver resections are still technically demanding, and a thorough case selection should be followed until the learning curve of major RLS is reached.

Although outcomes of the current study imply a safe and efficient application of RLS, it is important to interpret these results with respect to outcomes of laparoscopic liver surgery, which is currently considered the standard of care and the most used minimally invasive technique in liver surgery. A previous Dutch population-based study reported an increased use of laparoscopic liver surgery from 6% to 23% in 885 patients from 20 centers between 2011 and 2016.^15^ In contrast, building on the laparoscopic liver surgery experience, the nationwide use of RLS in the current study increased in an even faster pace. The conversion rate of 13%, median hospital stay of 5 days, severe morbidity of 8.6% and overall mortality of 1% with a R0 resection status of 89.4% in that previous study was rather comparable with the current outcomes of RLS. In addition, although selection criteria for laparoscopic liver resection in the Netherlands during implementation were limited to no need for vascular or biliary reconstruction and no need for a simultaneous anatomically major liver and colorectal resection, selection criteria for RLS as reported by the leading surgeons included the absence of centrally located lesions or indication for technically or anatomically major resection.^15^ We speculate that this careful inclusion may reflect the initial phase of adopting a new technique. Another analysis of 1131 patients after laparoscopic liver resection in 272 US centers reported a postoperative complication rate of 38.1%, mortality rate of 2.8%, and length of hospital stay of 5 days.^37^ Of note, outcomes from both laparoscopic liver surgery studies may seem to suggest the noninferiority of RLS as compared with the laparoscopic approach, yet differences in patient populations might still be present. Although several comparative studies have been published,^31^,^38^ future large series and randomized trials comparing RLS and laparoscopic liver surgery are needed to determine the added value of RLS in the current minimally invasive liver surgery practice.

Despite comparable outcomes of RLS and laparoscopic liver surgery in the current literature, the robotic technique might have several potential advantages as compared with the laparoscopic technique. Robotic instruments have an increased dexterity as compared with the conventional rigid laparoscopic instruments facilitating posterosuperior and major resections.^19^ Another major benefit of the robotic system is the ability to build an interactive visual interface, rather than a basic operating field, using customized software in which surgeons may be assisted by preoperative and/or intraoperative imaging such as intraoperative ultrasound and Indocyanine green fluorescence imaging during parenchymal resection.^39^ The robotic system may also ensure a fine and safe dissection of the hepatic pedicle through its delicate movements and endowrist instruments, allowing it to reach the hilum and the portal bifurcation easier, especially during anatomically major liver resection.^39^ Furthermore, robotic surgery is associated with less physical discomfort including fatigue as compared with open and laparoscopic surgery, given postural differences during laparoscopic, open and robotic surgery.^40^ The main drawbacks of the robotic approach include the higher cost and suboptimal availability of robotic systems as compared with laparoscopy. Results of the current study suggest that outcomes of RLS including postoperative complications, length of hospital stay, reoperations, and readmissions were favorable. These results imply that postoperative costs of liver resection could be lowered with the robotic approach. Also, with multiple new surgical robot devices currently developed, the hurdle to access and the costs of acquiring and maintaining a robotic system are expected to be lower. These benefits are likely to enhance the use of RLS globally, not just in high-volume centers but also in low-volume centers, but require confirmation in randomized trials of robotic versus laparoscopic liver surgery.

Interestingly, the survey results in the current study demonstrated that among robotic liver surgeons in the Netherlands, there is a considerable variation in training they completed before the start of their RLS program. This variation underlines the lack of a tailored and structured training program for RLS on national and international scale and, subsequently, the lack of standardized minimal requirements for a surgeon to initiate a RLS program in a center, as previously described.^41^ In the Netherlands, several training programs for minimally invasive laparoscopic and robotic pancreatic surgery (LAELAPS-1, 2, 3) were shown to be feasible and effective.^42^–^44^ Similarly, in the Netherlands, a training program for both technically and anatomically major laparoscopic liver surgery (LAELIVE) was initiated including detailed technique description and proctoring on-site. The aforementioned training programs support the feasibility and effectiveness of uniform and structured training programs in the field of minimally invasive HPB surgery and support the design of an international training program in RLS.

To our current understanding, this report is the only multicenter study investigating learning curves stratified for minor and major RLS.^45^ In trend analysis, the mean blood loss went down significantly for major resections with a range below 1000 mL after 40 to 50 consecutive procedures. On visual inspection of the blood loss CUSUM analysis, there is a clear peak at 33 RLS procedures followed by a second, final peak at 44 procedures. Furthermore, conversion diminished to 4%, although this difference was not significant. Most likely this can be attributed to the low event rate that comes with the low rate of conversion to begin with compared with laparoscopic liver resections. The proficiency learning phase was reached after 19 and 47 procedures for minor and major resections, respectively. Our results were within range of published data where 60 procedures were required for major RLS.^46^ Apart from the interpretation of the CUSUM analysis for blood loss, we acknowledge that a proficiency learning curve could change when the entire cohort completes more than 100 inclusions or when we could acquire data on operative times.

The current study has several limitations. First, the retrospective design might be accompanied with an inevitable risk of selection bias. Eligible patients have been selected specifically for RLS. Second, the annual volume of RLS per center was rather low over the study period, especially between 2014 and 2017. This shows that the adoption of RLS in the Netherlands is probably still in the early phase with acceptable outcomes and further extension might be observed in the next years. However, comparative data from other countries are lacking as this is the first nationwide series on RLS. Third, the current study used DHBA data with a fixed selection of collected variables. Specific information concerning operative time, intraoperative incidents, reason for conversion, cause of mortality were not registered in the DHBA and therefore could not be reported in the current study. Furthermore, data on postoperative complications and mortality in the DHBA are registered during hospital stay and (in case of earlier discharge) up until 30 days after surgery instead of 90 days. Our results should be interpreted carefully as assessing postoperative outcomes just at 30 days might miss a high number of major complications and deaths as compared with 90 days. Fourth, no data were available on costs of RLS, whereas previous studies highlighted its higher costs as compared with laparoscopic liver surgery.^38^ These data are needed although to determine cost-effectiveness of RLS. Fifth, the learning curves were assessed per center instead of per surgeon as specific data on which surgeon from each center performed the procedures was not available. Sixth, there may be some variation in pre-, intra-, and postoperative management strategies at the individual centers. Such data may be valuable to gain more insights in RLS outcome. However, the survey results demonstrated that surgical technique use for RLS was largely comparable.

## CONCLUSIONS

The present study showed that the nationwide implementation of RLS in the Netherlands has increased rapidly with currently one-tenth of all liver resections and one-fourth of all minimally invasive liver resections being performed robotically. RLS seems to be safe with promising outcomes for minor, technically major, and anatomically major RLS. Nevertheless, RLS remains complex and technically demanding and may benefit from the initiation of a structured and tailored (inter)national training program. Future large series or randomized trials comparing RLS and laparoscopic liver surgery are needed to determine the added value of RLS in the field of minimally invasive liver surgery.

## Supplementary Material
