# Supplementary material for: Implementation and Outcome of Robotic Liver Surgery in the Netherlands: A Nationwide Analysis
Source: Ann Surg. 2022 Jul 18;277(6):e1269–77. doi: 10.1097/SLA.0000000000005600 (PMC10174096; doi:10.1097/SLA.0000000000005600)
Supplement: Supplementary file 1 [file sla-277-e1269-s001.docx]

**ONLINE-ONLY SUPPLEMENTS**

**Supplement 1.** Survey to assess Surgical experience, Case Selection and Technique

1. Name
2. Institution
3. How many surgeons perform robotic liver surgery in your center?
4. How many fellows/residents are involved in the robotic liver surgery program in your center?
5. What form of training did you complete prior to the start of the robotic liver surgery program in your center?

*Please specify location and duration of training*

- Fellowship, ….
- Observership, …..
- Intuitive basic course, …..
- Intuitive specific course, …..
- Extern proctorship, …..
- Hands-on Course, …..
- Other, ….

1. What are the (relative) contraindications in your center for the use of the robotic approach in liver surgery?
2. Are you performing intraoperative ultrasound in all robotic liver resections? If so, do you use a laparoscopic or robotic approach?

- Yes, laparoscopic approach
- Yes, robotic approach
- No

1. Who performs intraoperative ultrasound in your center?
   - Radiologist
   - Surgeon
   - Both
2. Do you apply Indocyanine green (ICG) fluorescence imaging during robotic liver surgery?
   - Yes, 24 hours prior to surgery for tumor imaging
   - Yes, within 1 hour prior to surgery for biliary tract mapping
   - Yes, intra-operatively for liver perfusion assessment
   - No
   - Other, ….
3. Which Robotic system is primarily used in your center

- Yes, with da Vinci Xi, Intuitive Surgical
- Yes, with da Vinci X, Intuitive Surgical
- Yes, with da Vinci Si, Intuitive Surgical
- Yes, with da Vinci S, Intuitive Surgical
- Yes, with Senhance or TransEnterix
- Yes, other ….
- No

1. Do you have experience in other fields of robotic abdominal surgery? If so, specify the procedure and time of experience.
2. What robotic instruments do you use for minor robotic liver resection?

*Tick all that apply*

- - Vessel sealer
  - Robotic ultracision
  - Robotic bipolar device, *e.g.,* maryland
  - Monopolar scissors
  - Monopolar hook
  - Cadiere forceps
  - Extended grasper, *e.g.,* double-fenestrated grasper
  - Other:

1. What robotic instruments do you use for superficial liver parenchymal transection for major robotic liver resection?

*Tick all that apply*

- - Vessel sealer
  - Robotic ultracision
  - Robotic bipolar device, *e.g.,* maryland
  - Monopolar scissors
  - Monopolar hook
  - Cadiere forceps
  - Extended grasper, *e.g.,* double-fenestrated grasper
  - Other:

1. What robotic instruments do you use for deep liver parenchymal transection for major robotic liver resection?

*Tick all that apply*

- - Vessel sealer
  - Robotic ultracision
  - Robotic bipolar device, *e.g.,* maryland
  - Monopolar scissors
  - Monopolar hook
  - Cadiere forceps
  - Extended grasper, *e.g.,* double-fenestrated grasper
  - Other:

1. What robotic instruments do you use to divide vascular and biliary structures?

*Tick all that apply*

- - Vessel sealer
  - Robotic ultracision
  - Robotic bipolar device, *e.g.,* maryland
  - Monopolar scissors
  - Monopolar hook
  - Cadiere forceps
  - Extended grasper, *e.g.,* double-fenestrated grasper
  - Other:

1. How do you extract specimen after minor robotic liver surgery?
   - Through Pfannenstiel
   - Through Trocar site
   - Other, ….
2. How do you extract specimen after major robotic liver surgery?
   - Through Pfannenstiel
   - Through Trocar site
   - Other, ….

**Supplement 2.** Annual rate of open, laparoscopic, and robotic liver surgery in the nine Dutch liver surgical centers (2014-2020)


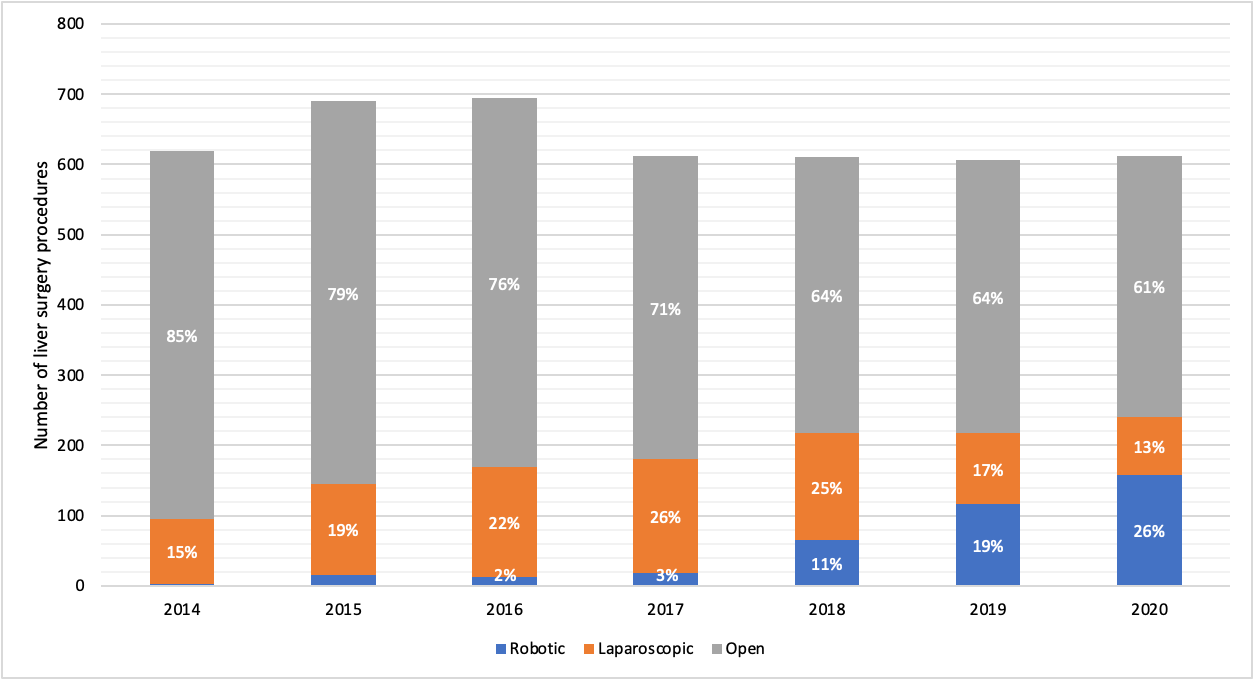


**Supplement 3.** Self-reported surgical selection factors for robotic liver surgery. Patient-related factors used by the nine participating Dutch centers to select patients for robotic liver surgery.

|  | **No centrally located tumors** | **No perihilar cholangiocarcinoma** | **No indication for technically or anatomically major liver resection** | **No indication for an extended hepatectomy** | **Small lesions** | **No major vascular or biliary duct involvement** | **No gallbladder carcinoma** | **No liver cirrhosis** | **No extensive previous abdominal surgery** |
| --- | --- | --- | --- | --- | --- | --- | --- | --- | --- |
| **Center 1** |  |  |  |  |  |  |  |  |  |
| **Center 2** |  |  |  |  |  |  |  |  |  |
| **Center 3** |  |  |  |  |  |  |  |  |  |
| **Center 4** |  |  |  |  |  |  |  |  |  |
| **Center 5** |  |  |  |  |  |  |  |  |  |
| **Center 6** |  |  |  |  |  |  |  |  |  |
| **Center 7** |  |  |  |  |  |  |  |  |  |
| **Center 8** |  |  |  |  |  |  |  |  |  |
| **Center 9** |  |  |  |  |  |  |  |  |  |

Green/dots= selection factor for RLS in respective center; Red/diagonal lines= no selection factor for RLS in respective center

**Supplement 4.** Self-reported robotic surgical technique in the nine Dutch liver surgical centers

**A B**


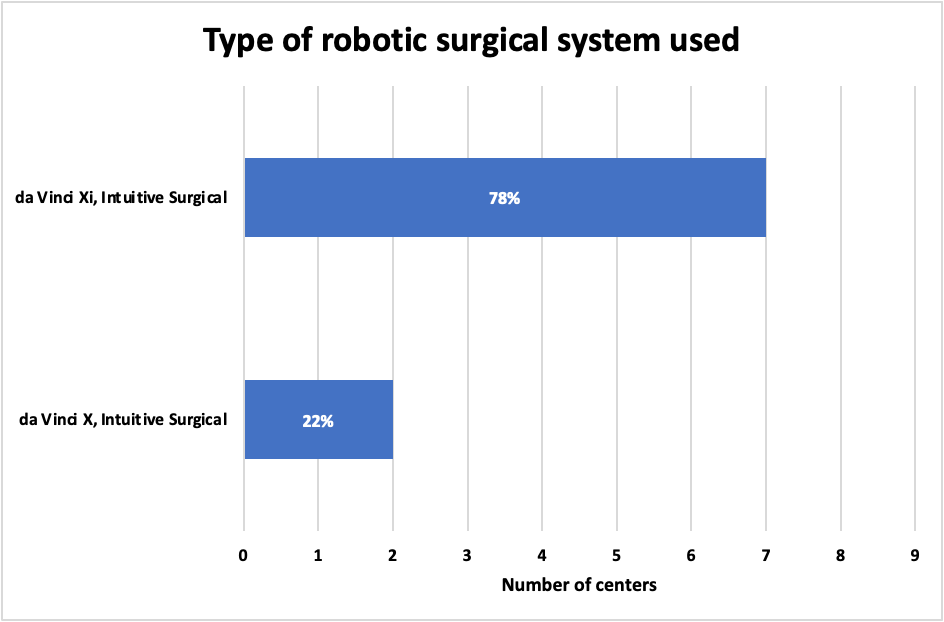

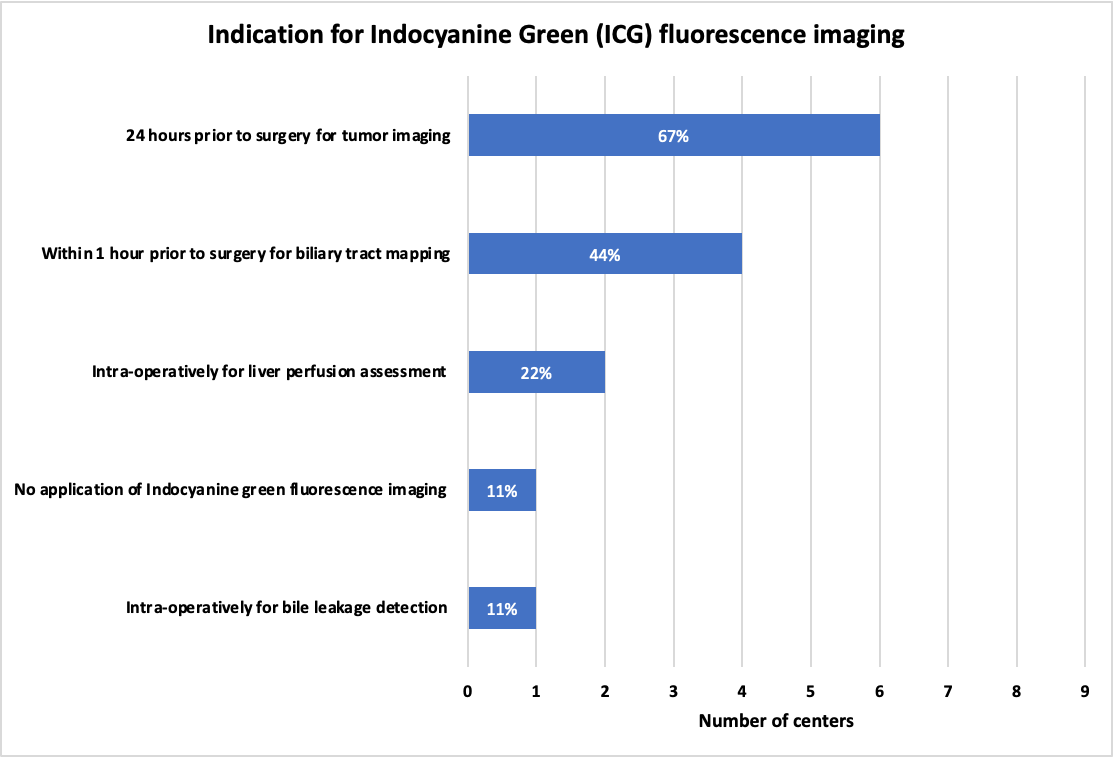


**C D**


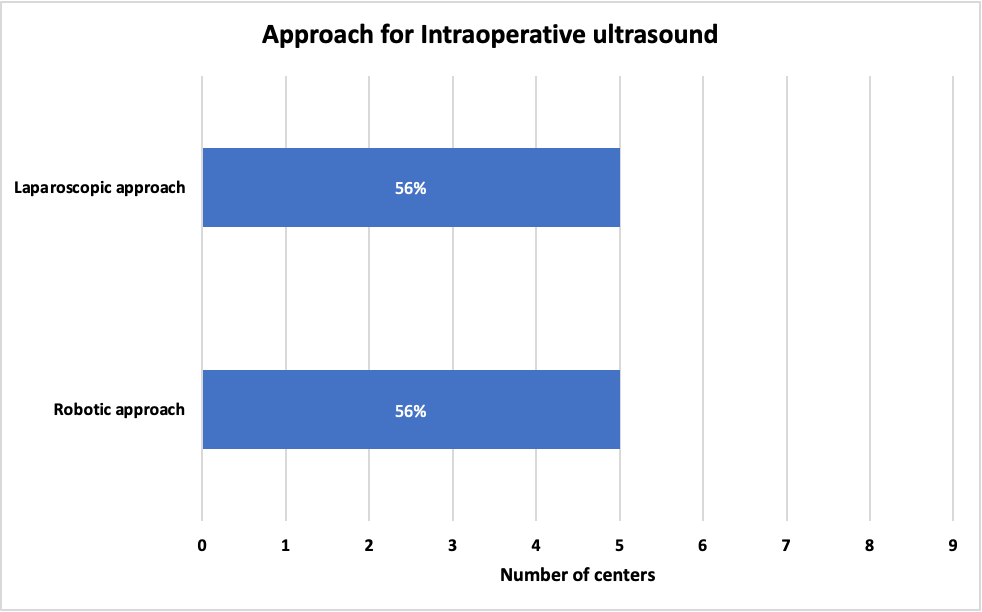

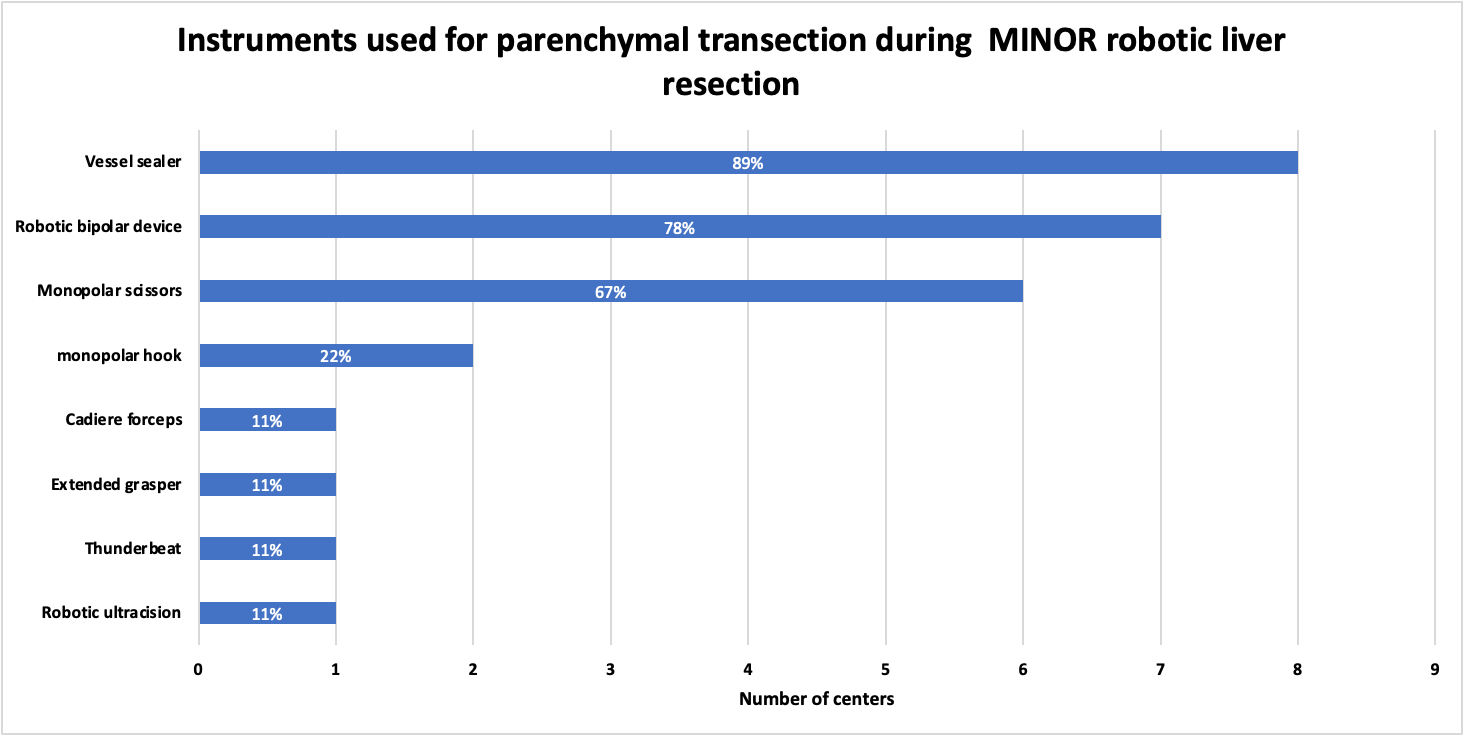


**E F**


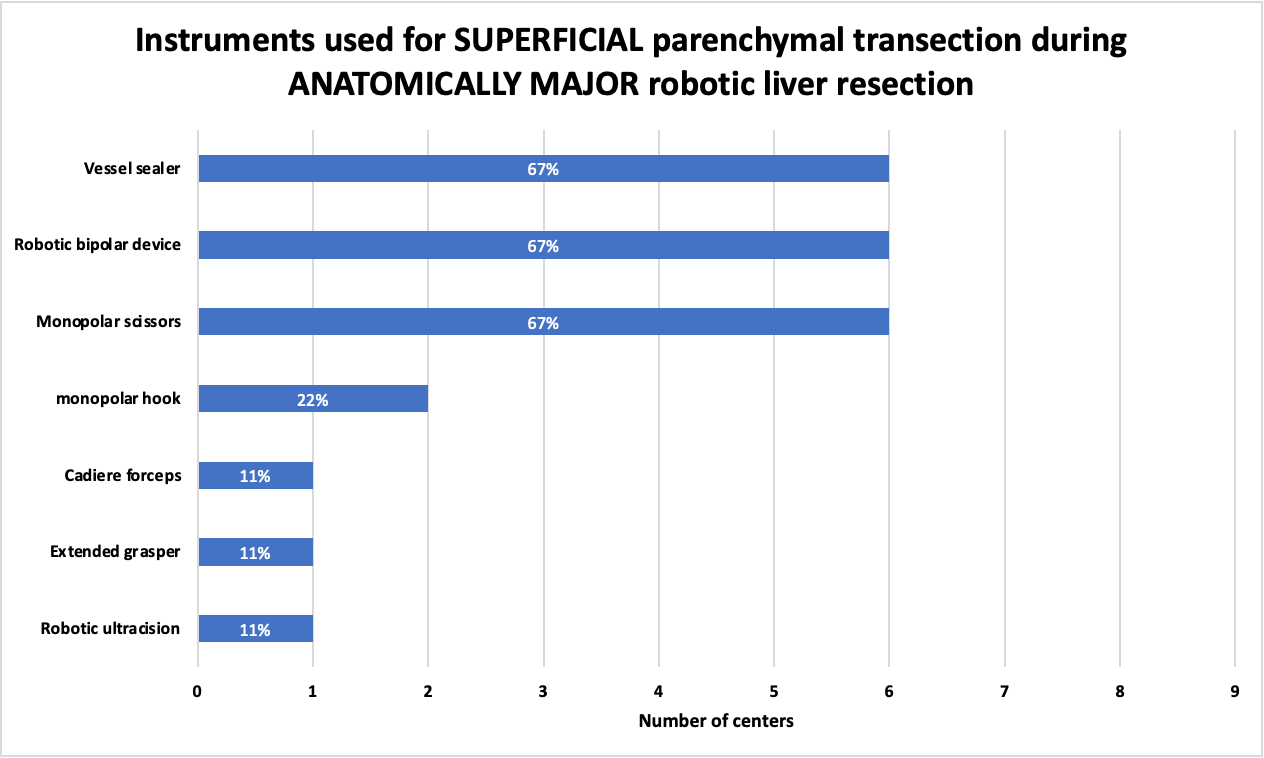

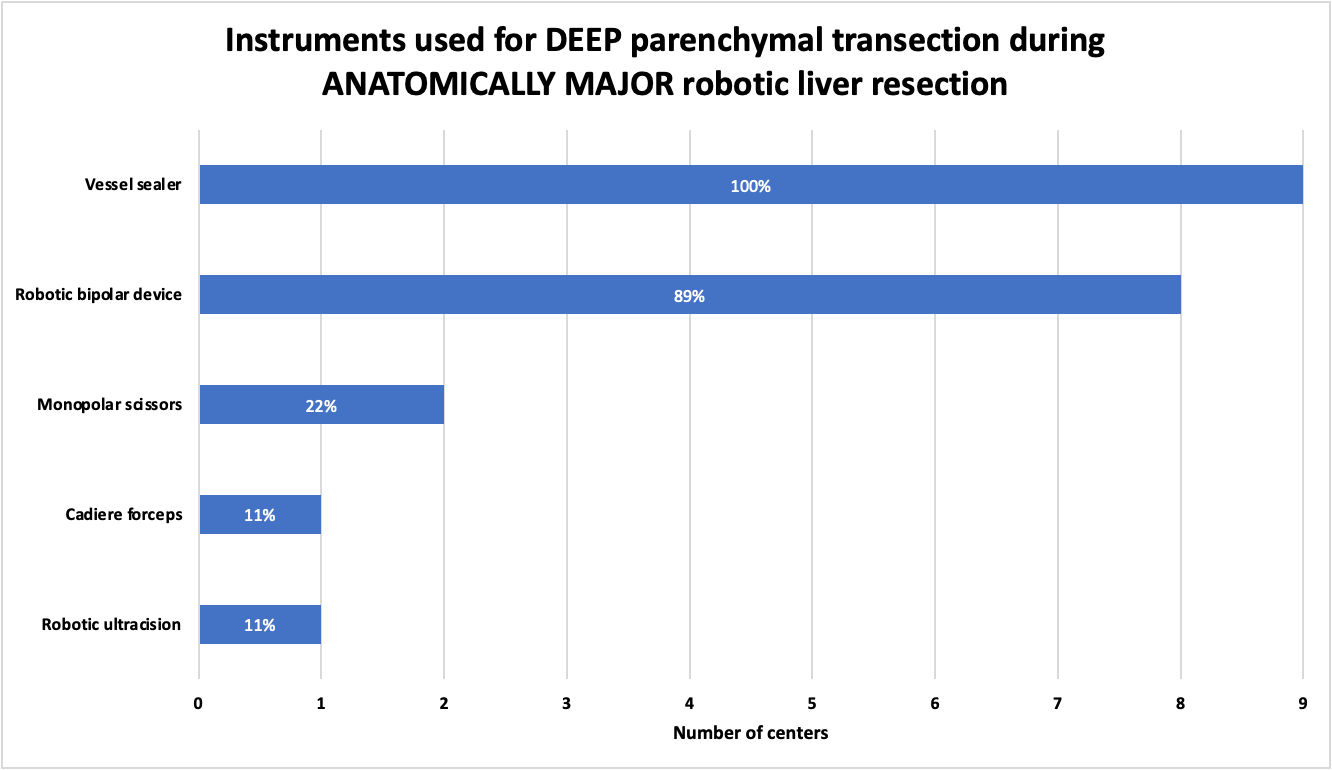


**G**


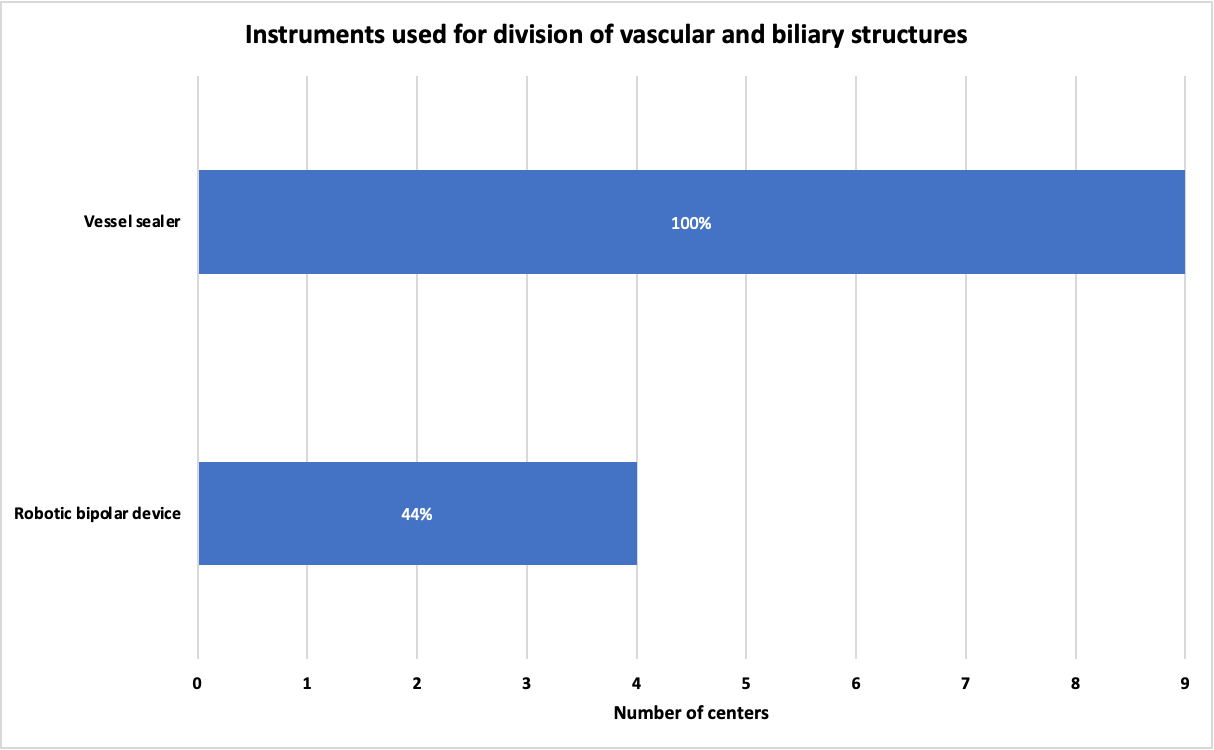


**Supplement 5.** Baseline characteristics of patients undergoing robotic liver surgery, stratified for type of surgery

|  | **All RLS**  **N=400** | **Minor RLS**  **N=207** | **Technically Major RLS**  **N= 141** | **Anatomically Major RLS**  **N=52** |
| --- | --- | --- | --- | --- |
| Age, years, median (IQR) | 64.0 (53.0-72.0) | 63.0 (50.0-72.0) | 65.0 (56.5-72.0) | 66.5 (53.3-71.8) |
| Sex, male (%) | 221 (55.3) | 105 (50.7) | 88 (62.4) | 28 (53.8) |
| BMI, kg/m^2^, median(IQR) | 25.9 (23.0-29.5) | 26.7 (23.4-30.8) | 24.9 (22.9-28.3) | 25.4 (23.2-28.8) |
| ASA grade  ASA 1 (%)  ASA 2 (%)  ASA 3 (%)  ASA 4 (%)  Charlson Comorbidity Index, median (IQR) | 33 (8.3)  253 (63.9)  108 (27.3)  2 (0.5)  3.0 (2.0-5.0) | 21 (10.2)  125 (61.0)  58 (28.3)  1 (0.5)  3.0 (1.5-6.0) | 10 (7.2)  93 (66.9)  35 (25.2)  1 (0.7)  3.0 (2.0-5.0) | 2 (3.8)  35 (67.3)  15 (28.8)  0  3 (2.0-5.0) |
| Neoadjuvant chemotherapy (%) | 66 (16.5) | 25 (12.1) | 28 (19.9) | 13 (25.0) |
| Cirrhosis (%) | 29 (7.2) | 18 (8.7) | 8 (5.7) | 3 (5.8) |
| Previous extrahepatic abdominal surgery (%) | 195 (50.4) | 98 (49.7) | 82 (59.4) | 15 (28.8) |
| Previous liver surgery (%) | 44 (11.0) | 20 (9.7) | 17 (12.1) | 7 (13.5) |
| Histological Diagnosis  CRLM (%)  HCC (%)  Cholangiocarcinoma (%)  Gallbladdercarcinoma (%)  Non-CRLM (%)  Other malignancy (%)  Benign (%) | 218 (55.2)  54 (13.5)  13 (3.3)  2 (0.5)  23 (5.8)  23 (5.8)  62 (15.5) | 99 (48.1)  29 (14.1)  8 (3.9)  2 (1.0)  14 (6.8)  12 (5.8)  42 (20.4) | 95 (68.3)  17 (12.2)  1 (0.7)  0  9 (6.5)  5 (3.6)  12 (8.6) | 24 (48.0)  8 (16.0)  4 (8.0)  0  0  6 (12.0)  8 (16.0) |
| Number of lesions, median (IQR) | 1 (1-2) | 1 (1-1) | 1 (1-2) | 1 (1-2) |
| Size of largest lesion, mm, median (IQR) | 27 (17-43) | 26 (18-45) | 25 (14-36) | 40 (26-65) |
| Distribution of lesions  Unilobar (%)  Bilobar (%) | 318 (86.2)  51 (13.8) | 171 (90.5)  18 (9.5) | 101 (78.9)  27 (21.1) | 46 (88.5)  6 (11.5) |
| Extent of resection  Wedge (%)  Segmentectomy (%)  Bisegmentectomy (%)  Trisegmentectomy  Left hemihepatectomy (%)  Right Hemihepactectomy (%)  Extended Right Hemihepatectomy (%)  Other anatomically major (%) | 177 (44.3)  77 (19.3)  94 (23.5)  5 (1.3)  18 (4.5)  26 (6.5)  2 (0.5)  1 (0.3) | 94 (45.4)  43 (20.8)  70 (33.8)  -  -  -  -  - | 83 (58.9)  34 (24.1)  24 (17.0)  -  -  -  -  - | -  -  -  5 (9.6)  18 (34.6)  26 (50.0)  2 (3.8)  1 (1.9) |

Values in parentheses are percentages unless mentioned otherwise. Percentages may not add up due to rounding and missing data. IQR = inter quartile range, BMI = body mass index, ASA = American Society of Anesthesiology, CRLM = colorectal liver metastasis, HCC = hepatocellular carcinoma

**Supplements 6.** Operative outcomes after robotic liver resections stratified for the Kawaguchi difficulty score.

|  | ***Group I***  **N=242** | ***Group II***  **N= 73** | ***Group III***  ***N=85*** |
| --- | --- | --- | --- |
| Blood loss (mL), median(IQR) | 100 (50-250) | 200 (50-425) | 400 (200-1200) |
| Conversion to laparotomy | 5 (2.1) | 5 (6.8) | 15 (17.6) |
| Postoperative complications | 40 (16.5) | 15 (20.5) | 21 (24.7) |
| Severe Postoperative complications | 14 (6.0) | 2 (2.9) | 11 (13.4) |
| Postoperative hospital stay (days), median(IQR)  Reoperation within 30 days | 3 (2-5)  6 (2.6) | 4 (3-6)  2 (2.9) | 5 (3-8)  2 (2.4) |
| Readmission within 30 days | 7 (2.9) | 1 (1.4) | 4 (4.9) |
| R0 resection in case of malignancy | 162 (85.7) | 46 (82.1) | 56 (77.8) |
| 30-day Mortality | 0 | 0 | 3 (3.7) |

Values in parentheses are percentages unless mentioned otherwise. Values in bold are considered statistically significant (P<0.017) after Bonferroni correction. RLS = Robotic Liver Surgery; IQR = inter quartile range

**Supplement 7.** Type of surgery and operative outcomes after robotic liver resections stratified for centers where the leading console surgeon completed a fellowship in minimally invasive liver surgery and centers where the leading console surgeon completed another type of previous training.

|  | **Procedures by surgeons after a fellowship minimally invasive liver surgery**  **N=130** | **Procedures by surgeons without a fellowship minimally invasive liver surgery**  **N= 270** | **P** |
| --- | --- | --- | --- |
| Type of surgery  Minor (%)  Technically Major (%)  Anatomically Major (%) | 51 (39.2)  54 (41.5)  25 (19.2) | 156 (57.8)  87 (32.2)  27 (10.0) | **0.001** |
| Blood loss (mL), median(IQR) | 200 (50-500) | 100 (50-300) | **0.009** |
| Conversion to laparotomy | 9 (6.9) | 16 (5.9) | 0.700 |
| Postoperative complications | 14 (10.8) | 62 (23.0) | **0.005** |
| Severe postoperative complications | 6 (4.6) | 21 (7.8) | 0.221 |
| Postoperative hospital stay (days), median(IQR) | 4 (2-5) | 4 (2-6) | 0.859 |
| Reoperation within 30 days | 2 (1.5) | 8 (3.0) | 0.379 |
| Readmission within 30 days | 3 (2.3) | 9 (3.3) | 0.607 |
| R0 resection in case of malignancy | 83 (82.2) | 181 (83.3) | 0.540 |
| 30-day mortality | 1 (0.8) | 2 (0.7) | 0.960 |

**Supplement 8.** Operative outcomes after robotic liver resections stratified for patient with and without liver cirrhosis

|  | **RLS patients with cirrhosis**  **N=29** | **RLS patients without cirrhosis**  **N= 371** | **P** |
| --- | --- | --- | --- |
| Blood loss (mL), median(IQR) | 200 (63-500) | 150 (50-350) | 0.369 |
| Conversion to laparotomy | 4 (13.8) | 21 (5.7) | 0.081 |
| Postoperative complications | 6 (20.7) | 70 (18.9) | 0.826 |
| Severe postoperative complications | 4 (13.8) | 23 (6.4) | 0.136 |
| CCI, mean(SD) | 39.5 (33.6) | 26.2 (14.2) | 0.069 |
| Postoperative hospital stay (days), median(IQR) | 4 (3-8) | 4 (2-5) | 0.190 |
| Reoperation within 30 days | 2 (6.9) | 8 (2.2) | 0.129 |
| Readmission within 30 days | 0 (0) | 12 (3.3) | 0.323 |
| R0 resection in case of malignancy | 25 (92.6) | 239 (82.4) | 0.391 |
| 30-day mortality | 2 (6.9) | 1 (0.3) | **<0.001** |

**Supplement 9.** Trends in length of hospital stay stratified for minor, technically and anatomically major robotic liver surgery.


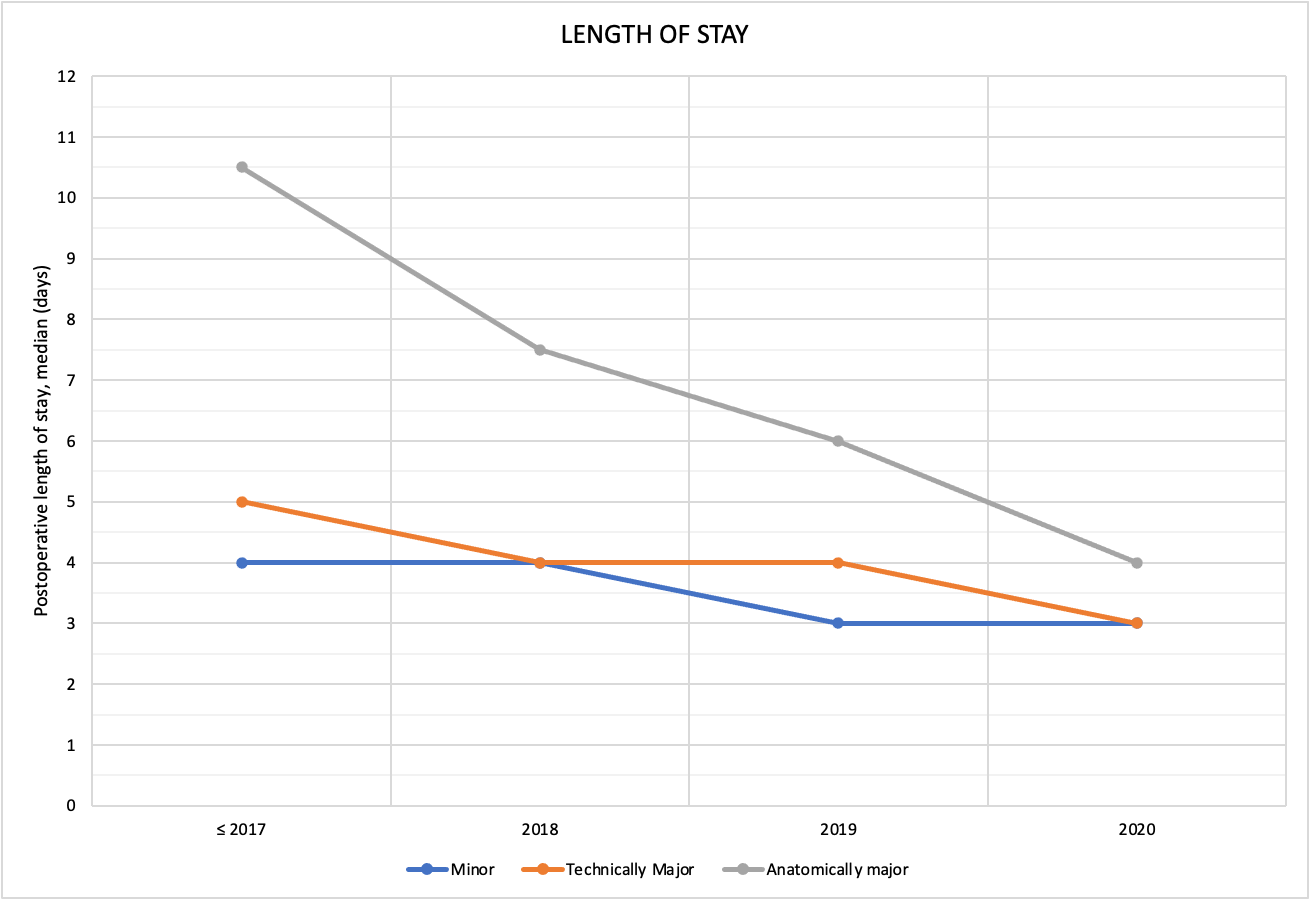


**Supplement 10.** Learning curve analysis in patients undergoing robotic liver surgery for blood loss.


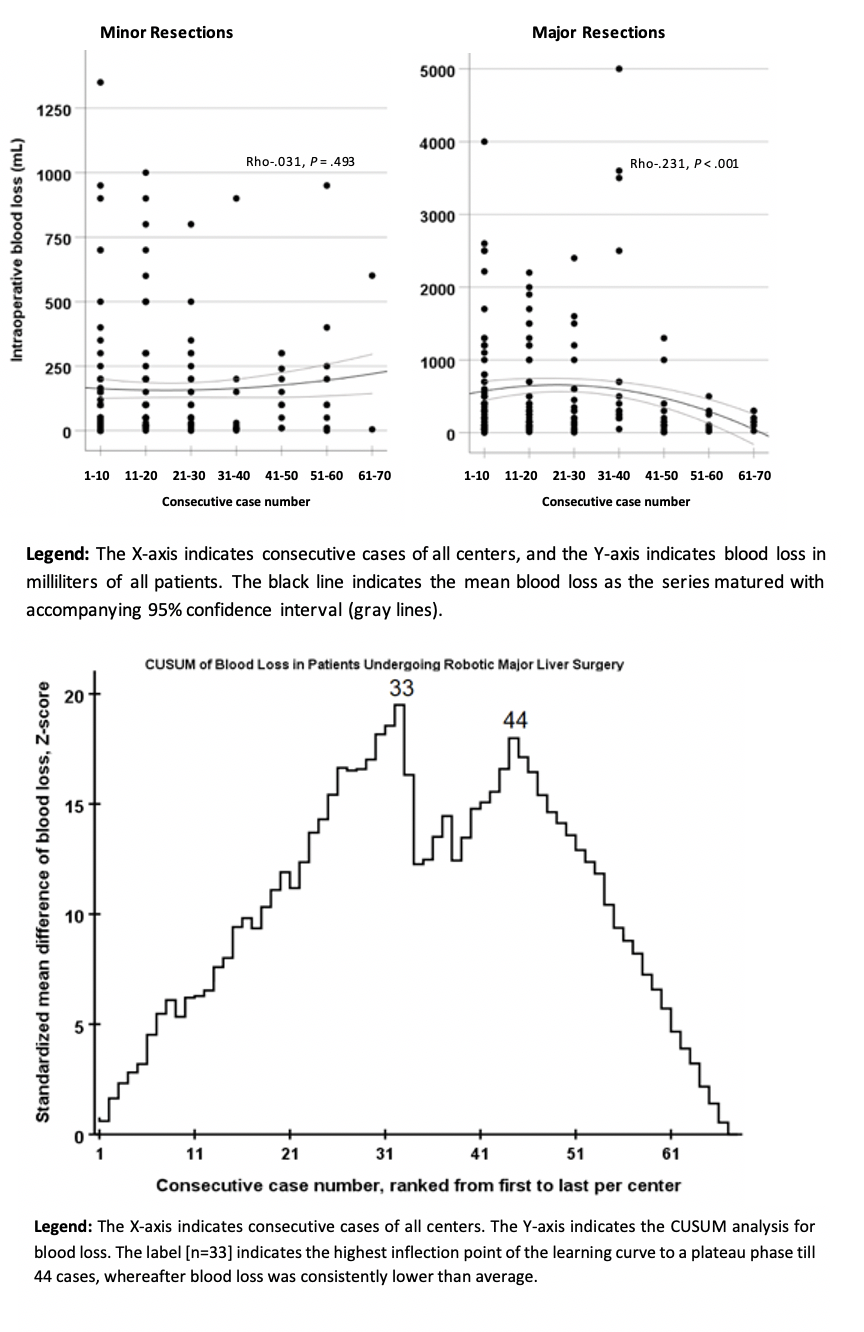


**Supplement 11.** Learning curve analysis in patients undergoing robotic liver surgery for length of stay.


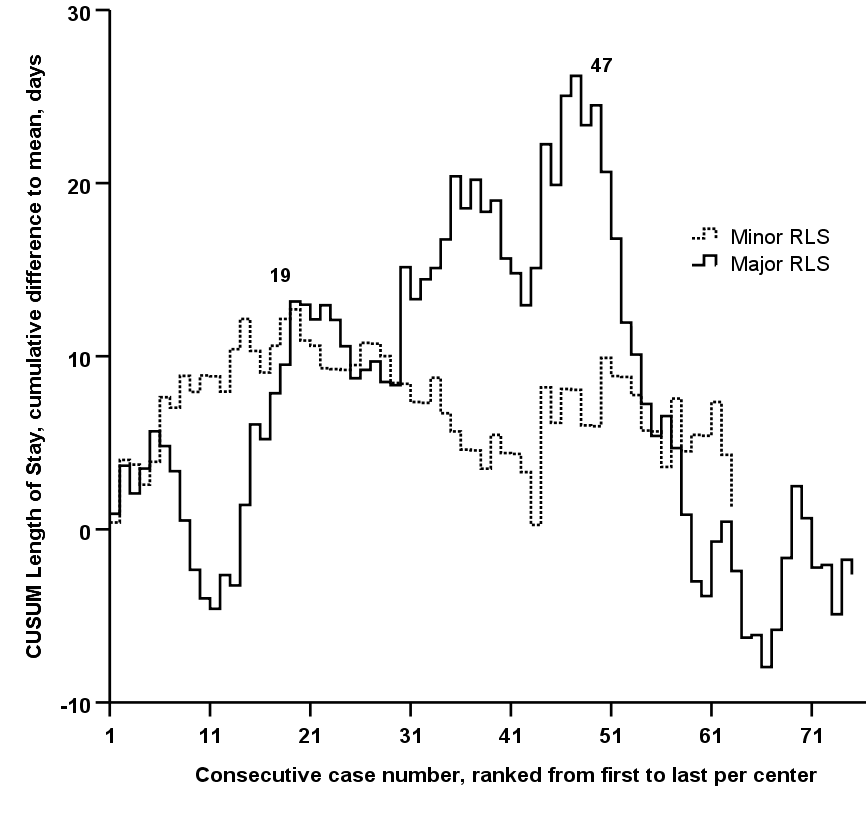


**Legend:** The X-axis indicates consecutive cases of all centers. The Y-axis indicates the CUSUM analysis for total length of stay. The label [n=19] indicates the highest inflection point of the learning curve for minor resections, the label [n=47] indicates the highest inflection point of the learning curve for major whereafter LOS was lower compared to before the inflection, *P*=.043.
